# Exploring the ocular microecology and its role in pterygium based on metagenomics

**DOI:** 10.1128/spectrum.01730-25

**Published:** 2025-10-13

**Authors:** Qiheng Yuan, Yiying Yang, Yangyang Shen, Bianjin Sun, Siwen Chen, Chengzhi Zheng, Yongliang Lou, Meiqin Zheng

**Affiliations:** 1 National Clinical Research Center for Ocular Diseases, Eye Hospital, Wenzhou Medical University, Wenzhou, China; 2 Wenzhou Key Laboratory of Sanitary Microbiology, Key Laboratory of Laboratory Medicine, Ministry of Education, School of Laboratory Medicine and Life Science, Wenzhou Medical University, Wenzhou, Zhejiang, China; 3 Nanyang Second People’s Hospital, NanYang, Henan, China; Université de Lorraine, Nancy, France

**Keywords:** pterygiums, ocular surface microbiome, pterygium tissue microbiome, metagenomic shotgun sequencing, variance analysis, virulence factor, antibiotic resistance genes

## Abstract

Pterygium is a chronic ocular surface condition marked by fibrovascular growth extending from the conjunctiva to the cornea. Emerging evidence suggests that microbial dysbiosis may play a role in its pathogenesis. To elucidate the microbial landscape associated with pterygium, we conducted metagenomic shotgun sequencing on conjunctival sac secretions from 24 patients with pterygium and 23 healthy controls, along with 19 pterygium tissue samples. We observed significantly higher microbial richness in the disease group, with distinct taxonomic profiles compared with healthy and tissue groups. Key species enriched in the disease group included *Microbacterium proteolyticum* and *Bacillus cereus*. Functional analyses revealed elevated bacterial motility, chemotaxis, and virulence genes, alongside a notable increase in antibiotic resistance genes such as tetB and AcrAB-TolC. In contrast, pterygium tissue samples showed limited microbial diversity and no detectable virulence or resistance genes. Importantly, the predominance of *Vibrio* phages in tissue samples, together with the frequent detection of their bacterial host *Vibrio diabolicus*, suggests a potential region-specific microbial risk factor, particularly relevant in coastal populations. These findings highlight distinct microbiome and functional profiles associated with pterygium, providing new insights into its pathogenesis and possible microbiome-based therapeutic targets.

**IMPORTANCE:**

Understanding how microbial communities contribute to ocular diseases is crucial for advancing both diagnostics and therapy. This study provides the first integrated comparison of healthy ocular surfaces, diseased ocular surfaces, and pterygium tissues, revealing distinct microbial signatures and functional disruptions. The enrichment of specific bacterial taxa, virulence factors, and antibiotic resistance genes in diseased eyes underscores their potential role in shaping local immunity and driving disease progression. Meanwhile, the discovery of distinct viral elements in pterygium tissue expands current understanding of its microecological complexity. These findings lay a theoretical foundation for the development of microbiome-informed diagnostic tools and novel therapeutic interventions for pterygium.

## INTRODUCTION

The Human Genome Project, launched by the National Institutes of Health (NIH) in 2008, revealed the vast diversity of human microbiota (1). These studies have significantly contributed to understanding the composition, pathogenicity, and physiological impact of various human microbiomes (2). To date, substantial progress has been made in studying the human microbiota, including gut, skin, oral, and urogenital microbiomes (3–8), offering insights into their role in health and disease. In recent years, increasing attention has been given to the ocular surface microbiota (OSM) and its potential involvement in both health and disease (9). As an emerging field, OSM research suggests a role in local and systemic immune responses (10, 11). Its composition is influenced by host factors, environmental exposures, and iatrogenic interventions, and it contributes to pathogen defense through niche competition and nutrient utilization, ultimately impacting various systemic diseases (12, 13).

Pterygium is a common ocular disease characterized by chronic inflammatory lesions triggered by external stimuli. It manifests as fibrovascular conjunctival tissue proliferation, which progressively invades the cornea. The condition typically originates in the nasal interpalpebral fissure but may occur bilaterally. In severe cases, corneal involvement extends to the pupillary zone, significantly impairing vision (14, 15). The overall prevalence of pterygium in China is approximately 9%, with incidence increasing with age. It is more common in rural populations than urban areas and is particularly prevalent among individuals engaged in prolonged outdoor activities, with males being more frequently affected than females (16–18). The disease is associated with multiple environmental risk factors, including ultraviolet radiation, viral infections, genetic susceptibility, immune dysregulation, aseptic inflammation, wind exposure, dust, and mechanical irritation (14, 15, 19–21). Currently, surgical excision remains the primary treatment; however, postoperative recurrence poses a significant challenge, necessitating strategies that address both lesion removal and recurrence prevention (14, 22). Recent studies suggest that alterations in local ocular immunity may contribute to pterygium development and progression, though the precise mechanisms remain unclear. Additionally, growing evidence indicates that microbiome dysbiosis can modulate host immunity and contribute to disease pathogenesis (23–26), highlighting the importance of investigating the microbiome’s role in pterygium.

Although previous research has demonstrated the significance of ocular surface microbiota in conditions, such as blepharitis, meibomian gland dysfunction, keratitis, and dry eye disease (27–31), studies specifically examining the ocular microbiota in pterygium remain limited. This study employs metagenomic shotgun sequencing to comprehensively profile the microbial communities associated with pterygium, including archaea, bacteria, fungi, and viruses, and to characterize their functional attributes. Furthermore, microbial antibiotic resistance and potential virulence factors are monitored. The study primarily focuses on comparing the ocular microbiota of the pterygium-affected ocular surface and deeper tissue with that of healthy controls. By elucidating the interactions among commensal microbiota and their influence on immune mechanisms and pterygium recurrence risk, this research aims to bridge the existing knowledge gap regarding the relationship between microbiota and pterygium.

## MATERIALS AND METHODS

### Participant recruitment

This study adhered to the principles of the Declaration of Helsinki and was approved by the Ethics Committee of the Eye Hospital, Wenzhou Medical University (Approval No. 2023-028-K-02). Informed consent was obtained from all participants. A total of 24 subjects were enrolled in the Disease group and 23 in the Normal group (17 normal ocular surface samples were collected by the research team in the early stage) (32), with mean ages of 65.38 ± 8.90 and 58.43 ± 10.03 years, respectively (Table S1). No significant differences were observed between the groups in terms of age and sex (*P* > 0.05) (Table 1), indicating that these factors did not influence subsequent analyses. All patients in the Disease group met the surgical treatment criteria and were older than 18 years. The Normal group had no history or clinical signs of pterygium. Additionally, Normal group participants had not worn contact lenses or experienced ocular surface disease or trauma in the past 6 months. They had not undergone ocular surgery or taken antibiotics within three months and had no history of systemic diseases or malignancies. Follow-up revealed no adverse reactions in any participants.

**TABLE 1:** Comparison of age and gender differences between the Disease group and the Normal group

Group	Patients with pterygium	Healthy controls	*P*-value
Male/female	8/16	9/14	0.0545
Age (years)	65.7 ± 8.5	58.4 ± 9.6	0.067

### Sample collection and DNA extraction

All pterygium tissue samples were obtained after standard preoperative aseptic preparation (during surgery) and immediately frozen at −80°C; this potential influence was considered during data interpretation. Conjunctival sac secretions were obtained using the Copan ESwab transport system (collect before preoperative disinfection). For unilateral cases, samples were taken from the affected eye, while for bilateral cases, they were collected from the surgical eye. In healthy participants, one eye was randomly selected for sampling. All samples were placed on ice before being transferred to −80°C for storage until DNA extraction. DNA was extracted using QIAGEN Pathogen Lysis Tubes L and the QIAamp UCP Pathogen Mini Kit. The concentration and quality of the extracted DNA were assessed via a DeNovix DS-11 spectrophotometer and 1% agarose gel electrophoresis.

### Metagenomic shotgun sequencing

Illumina sequencing libraries were prepared using the NEBNext Ultra DNA Kit. PCR products were purified with the AMPure XP system, and library size distribution was assessed using the Agilent 2100 Bioanalyzer. Library quantification was performed via real-time PCR. Sequencing was performed on the Illumina HiSeq X10 platform at Novogene Corporation in Beijing, China.

### Bioinformatics analysis

Raw sequencing data underwent quality control using FastQC v0.11.4 to assess sequence quality and Trimmomatic v0.32 to remove adapter and low-quality sequences (33, 34). Trimmed high-quality reads were then aligned to the human reference genome (hg19) using Bowtie2 v2.3.4.3 (35), and host-derived sequences were removed to obtain clean microbial reads. MEGAHIT v1.2.9 was employed for metagenome assembly of high-quality microbial sequences (36), followed by gene prediction on assembled contigs using Prodigal v2.6.3 (37). Taxonomic classification of the non-redundant gene set was performed with Kraken 2 v2.1.3 (38). HUMAnN3 v3.0.0 was used to predict microbial gene families and metabolic pathway abundances (39). Taxonomic composition was further analyzed by submitting classification outputs to Megan (40, 41). When visualizing the relative abundance of species, we selected the top 10 phyla, the top 20 genera and species for visualization, and all other categories were placed under “Others.” For functional annotation, non-redundant gene sequences were mapped to the KEGG, COG, and the Carbohydrate-Active enZYmes (CAZy) reference databases. DIAMOND was used to align non-redundant amino acid sequences to CAZy database (42), while KAAS was employed for KEGG-based functional prediction. Virulence factors in the ocular surface microbiome were identified using the VFDB database (43), and ARGs-OAP v2.0 was applied to determine the abundance of antibiotic resistance genes (ARGs) (44, 45).

### Data processing and statistical analysis

Statistical analyses were performed using R v4.3.2. The vegan package was used to generate rarefaction curves and calculate Shannon and Simpson indices for α-diversity assessment. Principal coordinate analysis (PCoA) significance was analyzed using vegan, and Bray-Curtis distances were computed. The Wilcoxon rank-sum test was applied to compare Shannon and Simpson indices between the Disease and Normal groups, as well as between the Disease and Tissue groups. Bray-Curtis dissimilarity was used to estimate β-diversity between these groups, and PCoA was conducted for visualization. Permutational multivariate analysis of variance (PERMANOVA) was employed to assess differences in Bray-Curtis distances among groups. The Kruskal-Wallis test identified microbial taxa and functional pathways with significant differences. Linear discriminant analysis (LDA) effect size (LEfSe) was used to determine potential biomarkers distinguishing the Disease and Normal groups, as well as the Disease and Tissue groups (46). At the species level, the RandomForest package evaluated the classification contribution of different taxa. SparCC was used for correlation analysis of microbial genera (47, 48). Finally, heatmaps were generated using the Pheatmap package to visualize relationships among features in the data set (49).

## RESULTS

### Operational taxonomic unit (OTU) clustering and alpha diversity analysis of the ocular surface microbiome

**Fig 1:**
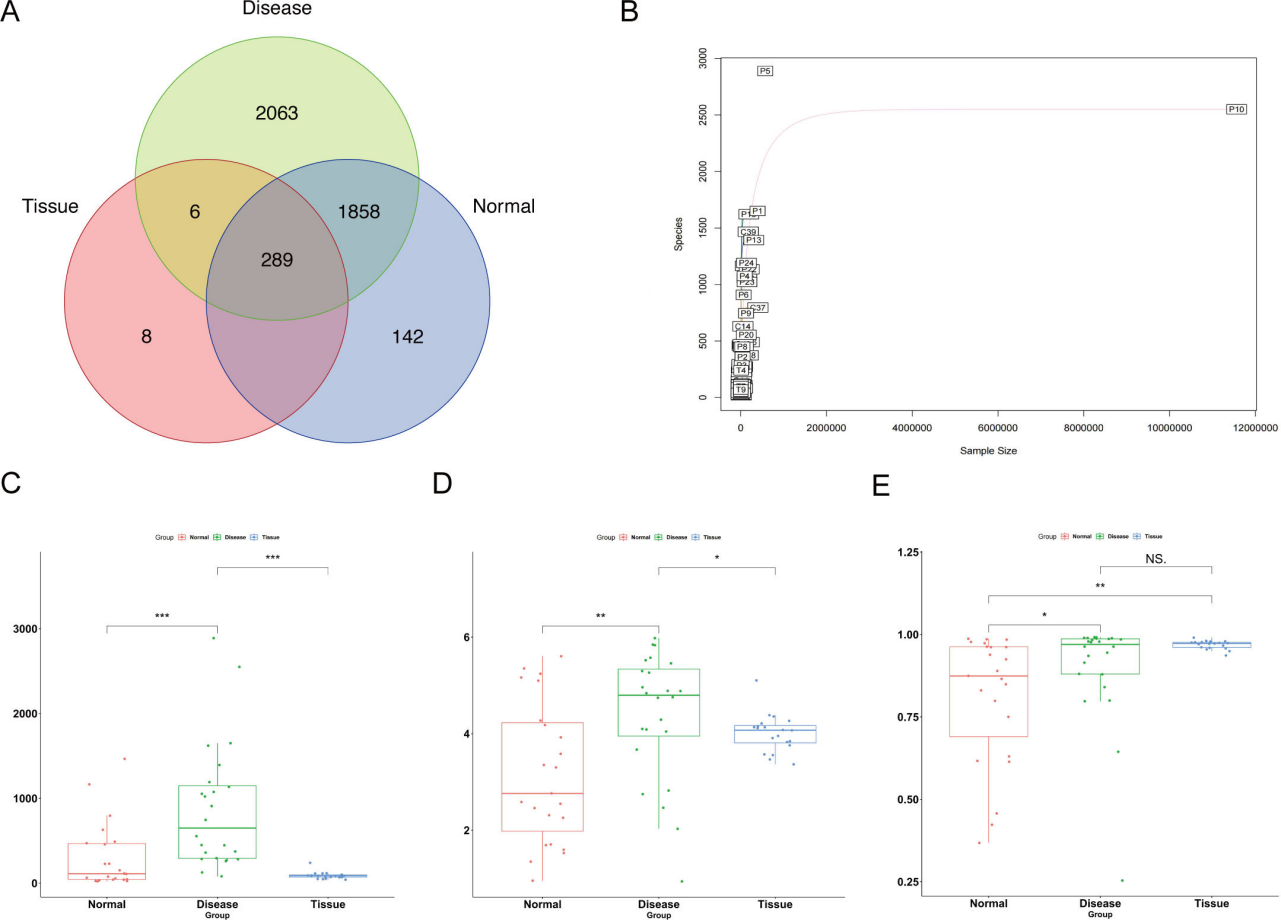
Analysis of ocular surface microbial diversity in three groups. (**A**) Venn diagram of OTU clustering analysis; (**B**) rarefaction curves of the samples; (**C**) microbial species abundance differences; (**D**) alpha diversity assessed by the Shannon index; (**E**) alpha diversity assessed by the Simpson index. *P < 0.05, **P < 0.01, and ***P < 0.001.

OTU clustering identified 4,366 distinct OTUs across the three groups. Venn diagram analysis showed that the Disease, Normal, and Tissue groups contained 4,210, 2,289, and 303 OTUs, respectively, with 289 shared among all groups. The Disease and Normal groups shared 2,147 OTUs, while the Disease and Tissue groups had 295 in common. The OTU count in the Tissue group was significantly lower than that in the Disease group (Fig. 1A).

Rarefaction curves flattened with increasing sequencing depth, indicating that the sequencing adequately captured species diversity (Fig. 1B). Alpha diversity analysis revealed a significantly higher microbial abundance in the Disease group compared with the other two groups. Moreover, the Shannon and Simpson indices were significantly higher in the Disease group than in the Normal group, and the Shannon index exceeded that of the Tissue group (Fig. 1C through E).

### Beta diversity analysis of Bray-Curtis dissimilarity sums in ocular surface difference groups

**Fig 2:**
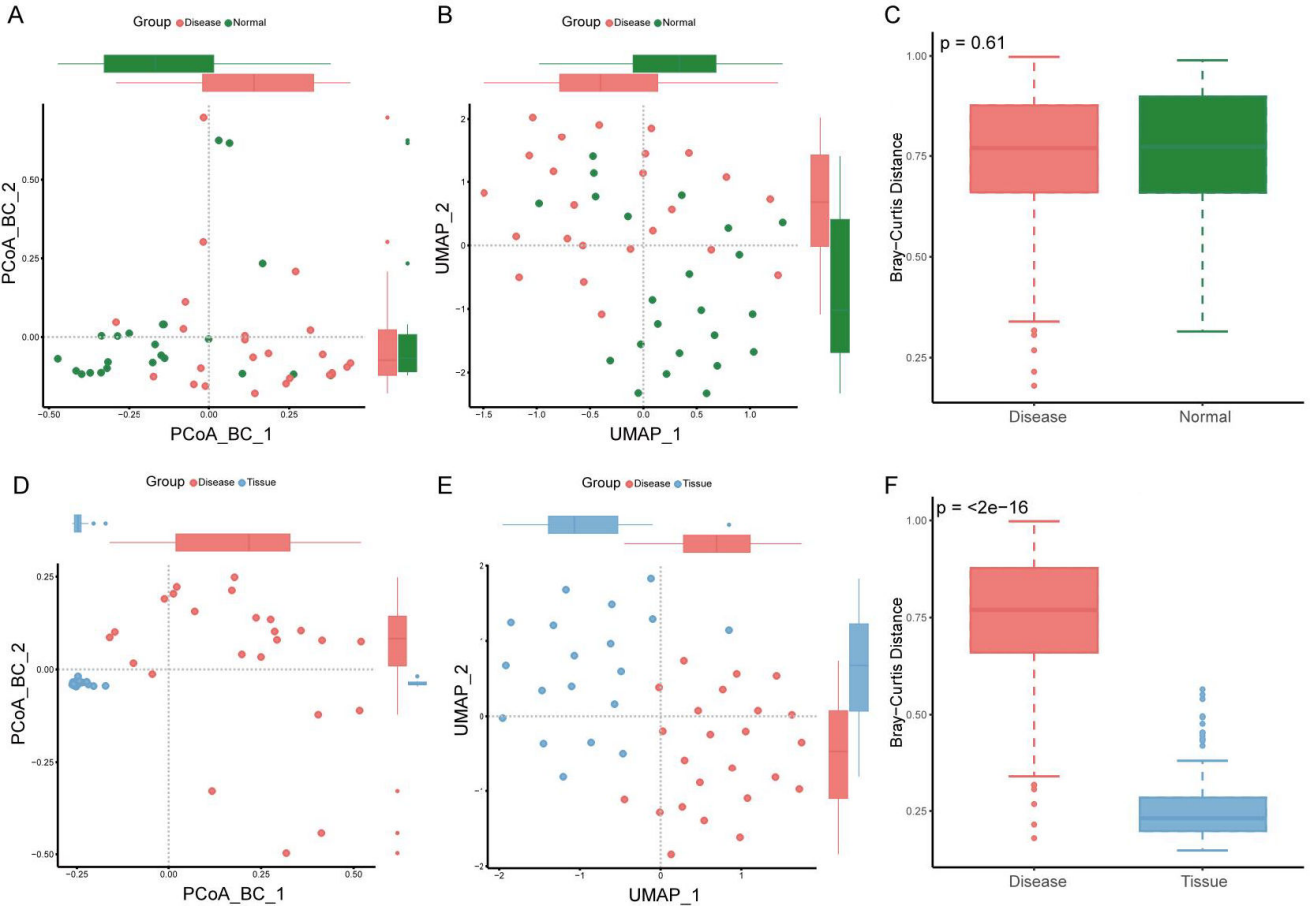
Beta diversity analysis, PCoA and UMAP plots based on Bray-Curtis distance. (**A–C**) Beta diversity between healthy subjects (Normal group) and pterygium patients (Disease group); (**D–F**) beta diversity between the ocular surface (Disease group) and pterygium tissue (Tissue group). PCoA, principal coordinates analysis, UMAP, uniform manifold approximation and projection.

Beta diversity analysis based on Bray-Curtis dissimilarity was conducted to assess differences in ocular surface microbiota between pterygium patients and healthy controls, as well as between the ocular surface and tissue of pterygium patients. Dimensionality reduction and visualization of distance matrices were performed using principal components analysis (PCA), PCoA, non-metric multidimensional scaling (NMDS), t-distributed stochastic neighbor embedding (TSNE), and uniform manifold approximation and projection (UMAP). The results showed that most samples from pterygium patients and healthy controls clustered together, while ocular surface and tissue samples formed separate clusters. PERMANOVA analysis indicated no significant difference in beta diversity between the Disease and Normal groups (Fig. 2A through C; Fig. S1), whereas a significant difference was observed between the ocular surface and tissue groups (Fig. 2D through F; Fig. S2).

### Kingdom-level species composition of the ocular surface microbiome

**Fig 3:**
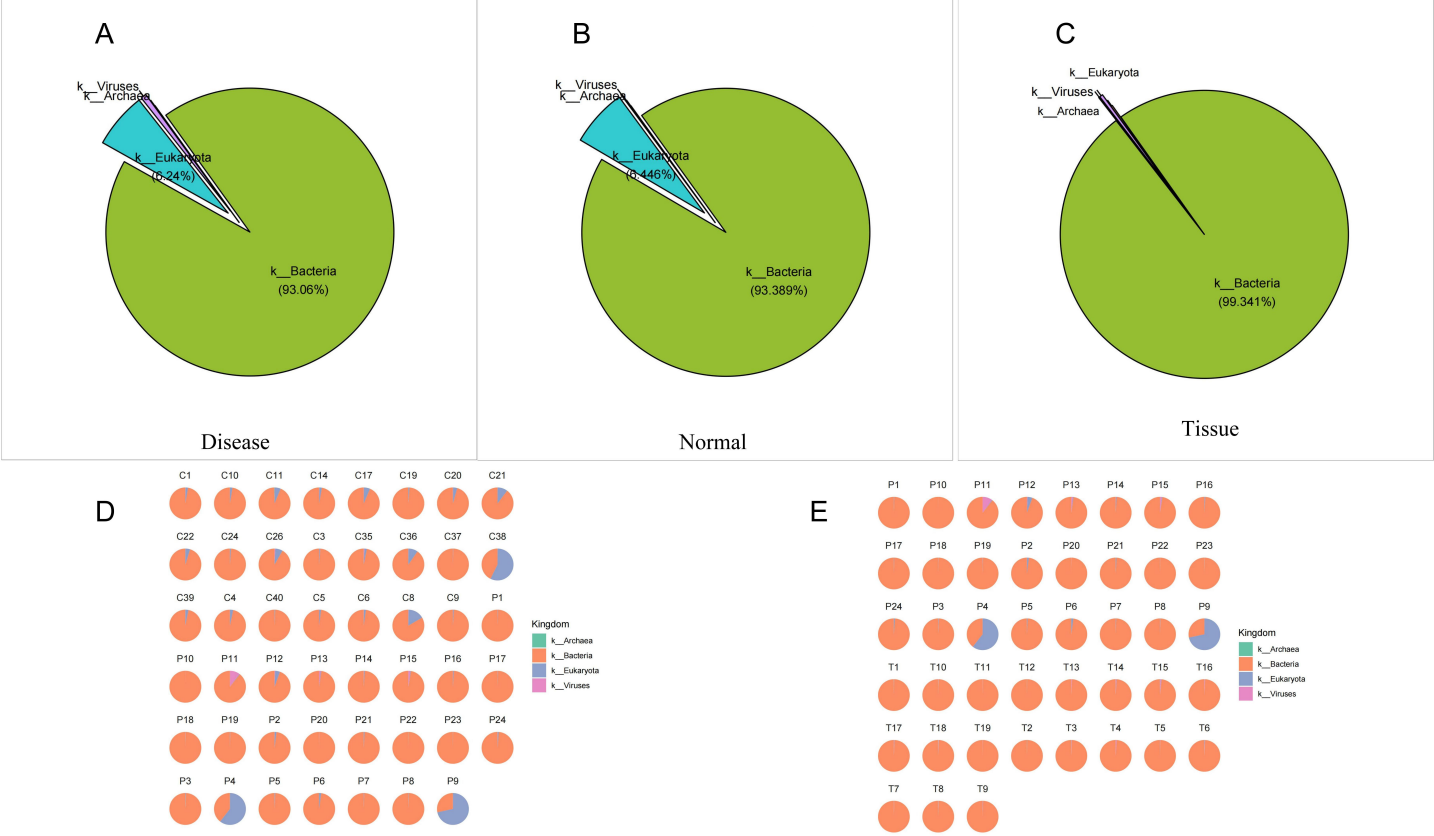
Taxonomic composition at the phylum level across all samples. Pie charts illustrate the average relative abundance of bacteria, archaea, fungi, and viruses in the ocular surface of pterygium patients (Disease group, **A**), healthy subjects (Normal group, **B**), and pterygium tissue (Tissue group, **C**). Average abundance distribution of individual samples in the Normal and Disease groups (**D**). Average abundance distribution of individual samples in the Disease and Tissue groups (**E**).

Taxonomic analysis revealed that over 90% of sequences were bacterial, while fungi, archaea, and viruses were detected at low abundances. Both the Disease and Normal groups exhibited a bacteria-dominated ocular microbiome, indicating that pterygium did not alter bacterial predominance. Bacteria and fungi were present in all participants, whereas archaea and viruses appeared in only a few samples. Notably, archaea were more prevalent in healthy samples but less common in pterygium cases, suggesting that the ocular environment in pterygium may be unfavorable for archaea survival or that archaea are outcompeted within the microbial community (Fig. 3).

### Phylum-level composition of the ocular surface microbiome

**Fig 4:**
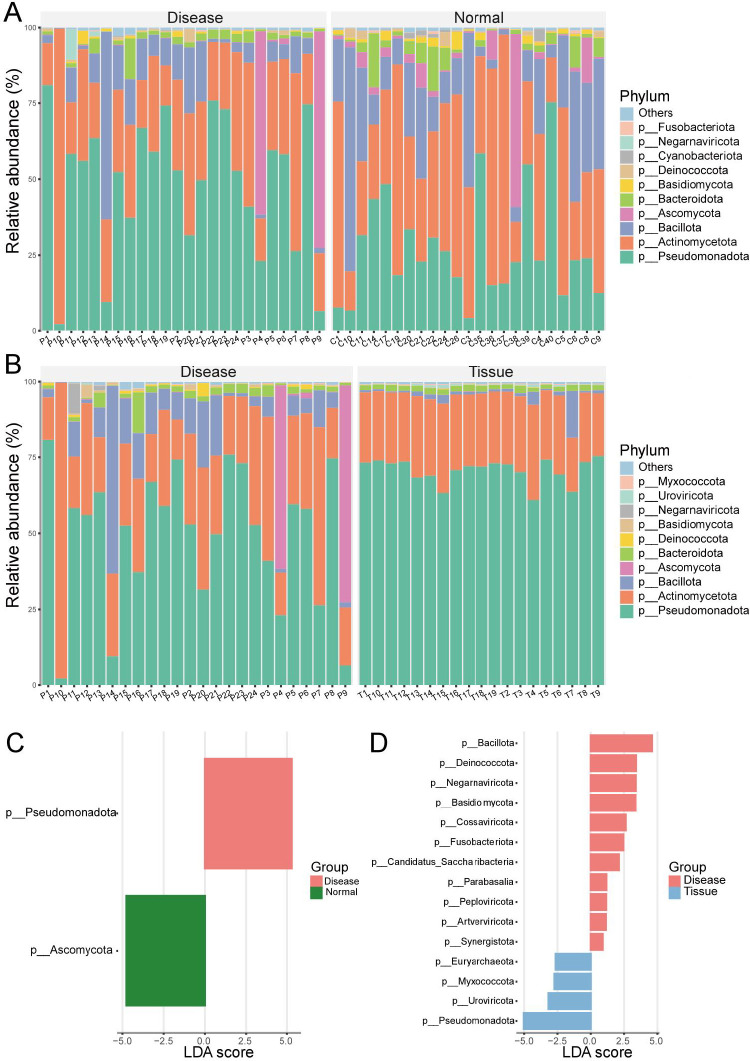
Phylum-level microbial abundance and differences. (**A**) Phyla with higher abundance in the Disease group compared with the Normal group. (**B**) Phyla with higher abundance in the Disease group compared with the Tissue group. (**C**) Differentially abundant phyla between the Disease and Normal groups. (**D**) Differentially abundant phyla between the Disease and Tissue groups.

At the phylum level, 74 bacterial phyla were identified in both the Disease (pterygium) and Normal groups. Figure 4A illustrates the top 10 phyla. The ocular microbiomes of both groups were dominated by *Pseudomonadota*, *Actinomycetota*, *Bacillota*, and *Bacteroidota*, while fungal and viral abundances remained low. LEfSe analysis identified two potential biomarkers: *Ascomycota* in the Normal group and *Pseudomonadota* in the Disease group (Fig. 4C).

Comparison between the Disease and Tissue groups revealed 72 bacterial phyla. The top 10 phyla are shown in Fig. 4B. Both groups were predominantly composed of *Pseudomonadota* and *Actinomycetota*, with minimal fungal and viral presence. LEfSe analysis identified 15 potential biomarkers: 11, including *Bacillota* and *Deinococcota*, were enriched in the Disease group, while 4, including *Pseudomonadota* and Uroviricota, were predominant in the Tissue group (Fig. 4D).

### Genus-level composition of the ocular surface microbiome

**Fig 5:**
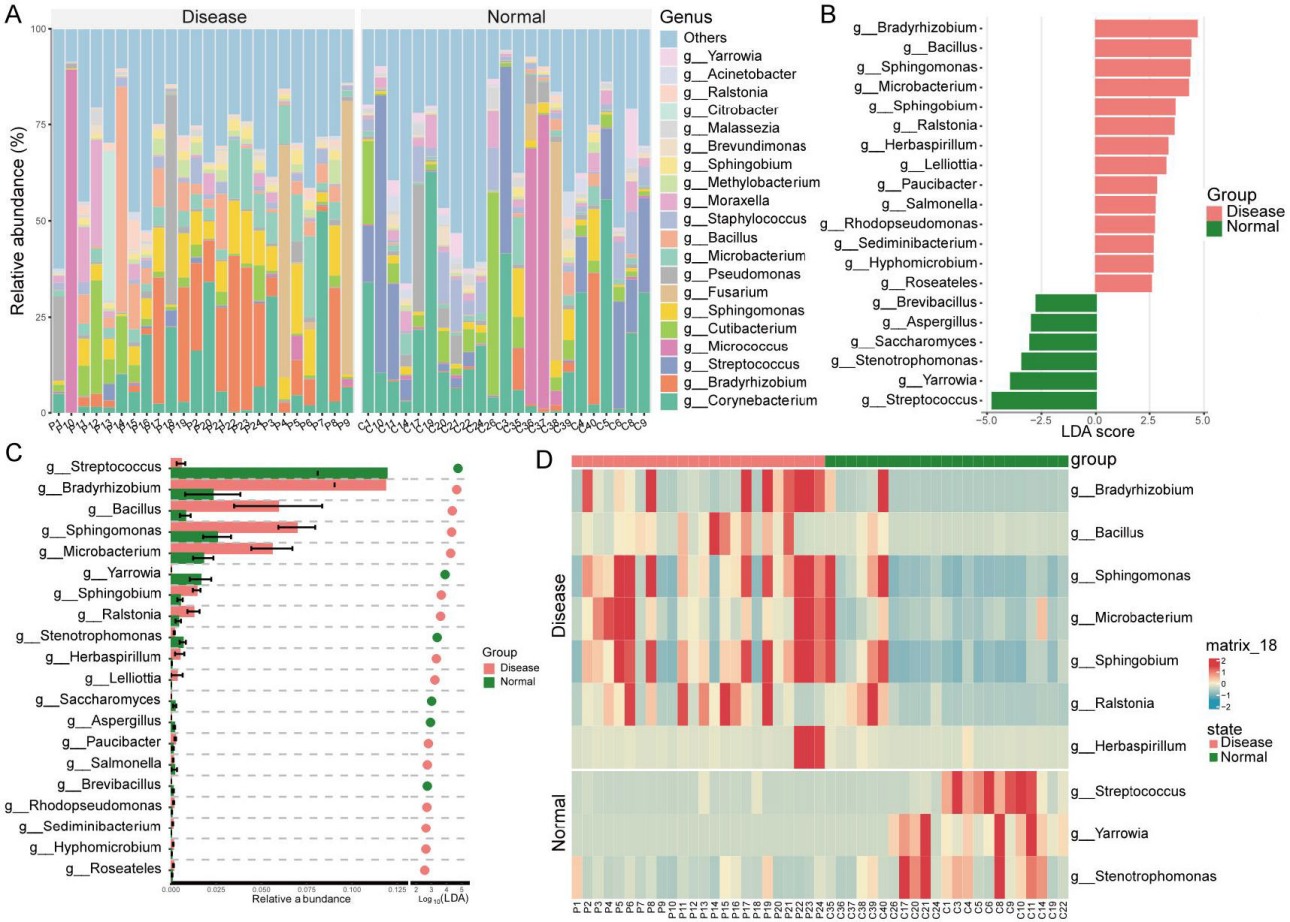
Genus-level microbial abundance and differences between the Disease and Normal groups Genera with higher abundance in the Disease and Normal groups. (**A**) Genus with higher abundance in the Disease group compared with the Normal group. (**B and C**) Differentially abundant genera between the Disease and Normal groups. (**D**) Heatmap showing the top 10 differentially abundant genera with the highest relative abundance across all samples in both groups.

At the genus level, 2,338 genera were identified across the Disease and Normal groups. Figure 8A presents the 20 most abundant genera. The Disease group was dominated by *Bradyrhizobium*, *Corynebacterium*, *Sphingomonas*, and *Microbacterium*, while the Normal group primarily consisted of Corynebacterium, *Cutibacterium*, and *Streptococcus*. Viral and fungal abundances were low in both groups, with *Yarrowia* and *Acinetobacter* among the other relatively abundant genera (Fig. 5A).

Differential and LEfSe analyzes identified 20 potential biomarkers. Compared with the Normal group, 14 genera, including *Bradyrhizobium*, *Bacillus*, and *Sphingomonas*, showed significantly higher relative abundances in the Disease group, making them potential biomarkers. Conversely, *Streptococcus* and *Yarrowia*, among six genera, were significantly enriched in the Normal group (Fig. 5B and C). Heatmap analysis further revealed that *Streptococcus*, *Yarrowia*, and *Stenotrophomonas* were more abundant in the Normal group, while *Bradyrhizobium*, *Bacillus*, and five other genera were elevated in the Disease group (Fig. 5D).

### Genus-level composition of the ocular surface microbiome in disease and tissue groups

**Fig 6:**
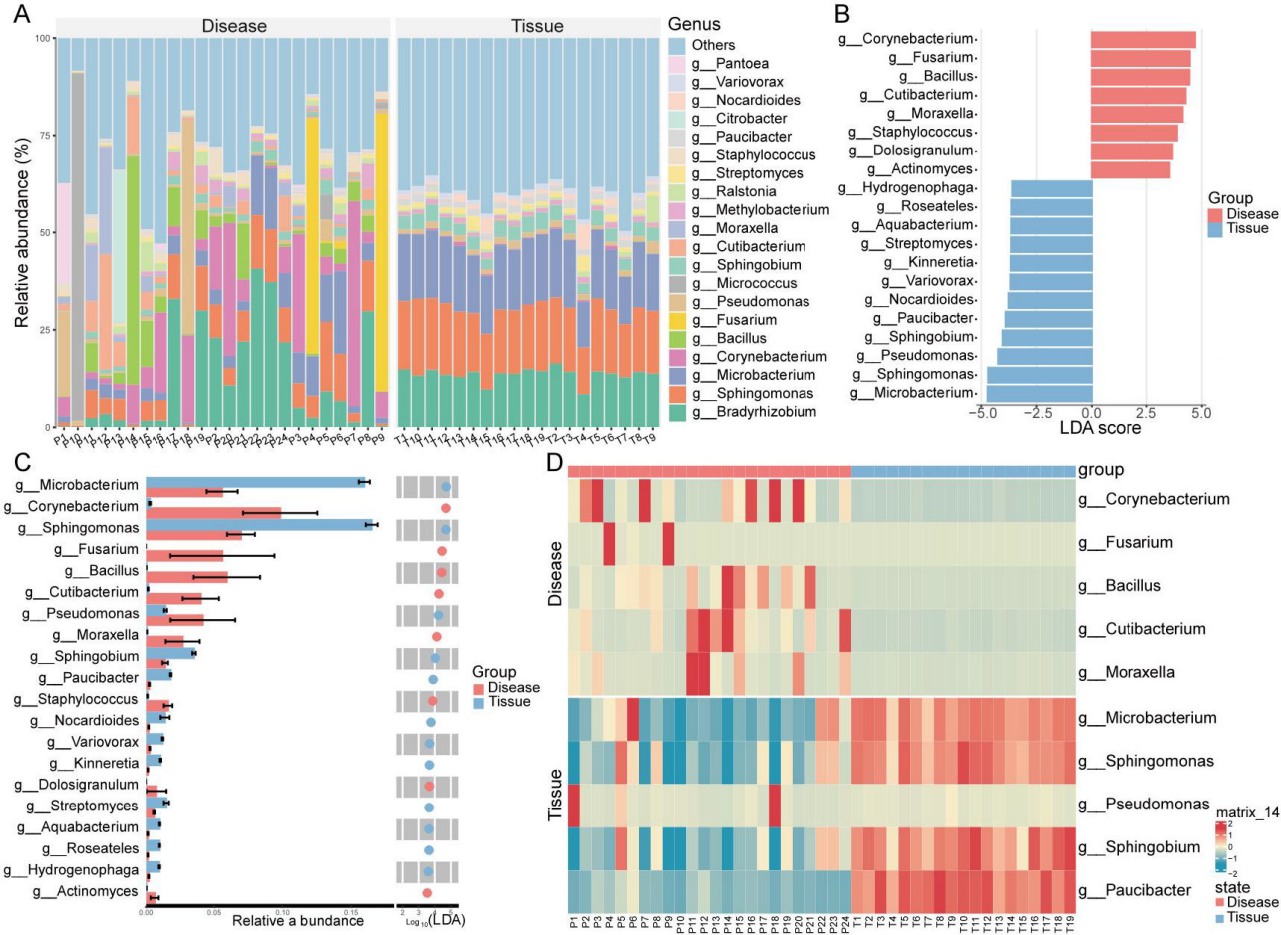
Genus-level microbial abundance and differences between the Disease and Tissue groups. Genera with higher abundance in the Disease and Tissue groups. (**A**) Genus with higher abundance in the Disease group compared with the Tiuuse group. (**B and C**) Differentially abundant genera and potential biomarkers between the Disease and Tissue groups. (**D**) Heatmap showing the top 10 differentially abundant genera with the highest relative abundance in both groups.

A total of 2,283 genera were identified across the Disease and Tissue groups. Figure 6 presents the 20 most abundant genera. The Tissue group was dominated by *Microbacterium*, *Sphingomonas*, and *Bradyrhizobium*, with minimal viral and fungal presence. Other genera with relatively high abundance included *Pantoea* and *Variovorax* (Fig. 6A).

Differential and LEfSe analyzes identified 20 potential biomarkers. Eight genera, including *Corynebacterium*, *Fusarium*, and *Bacillus*, were significantly enriched in the Disease group. In contrast, 12 genera, such as *Microbacterium*, *Sphingomonas*, and *Pseudomonas*, were more abundant in the Tissue group (Fig. 6B and C). Heatmap analysis further showed that *Microbacterium*, *Sphingomonas, and Pseudomonas* were significantly elevated in the Tissue group, while *Moraxella*, *Cutibacterium, and Bacillus* were enriched in the Disease group (Fig. 6D).

### Species-level composition of the ocular surface microbiome

**Fig 7:**
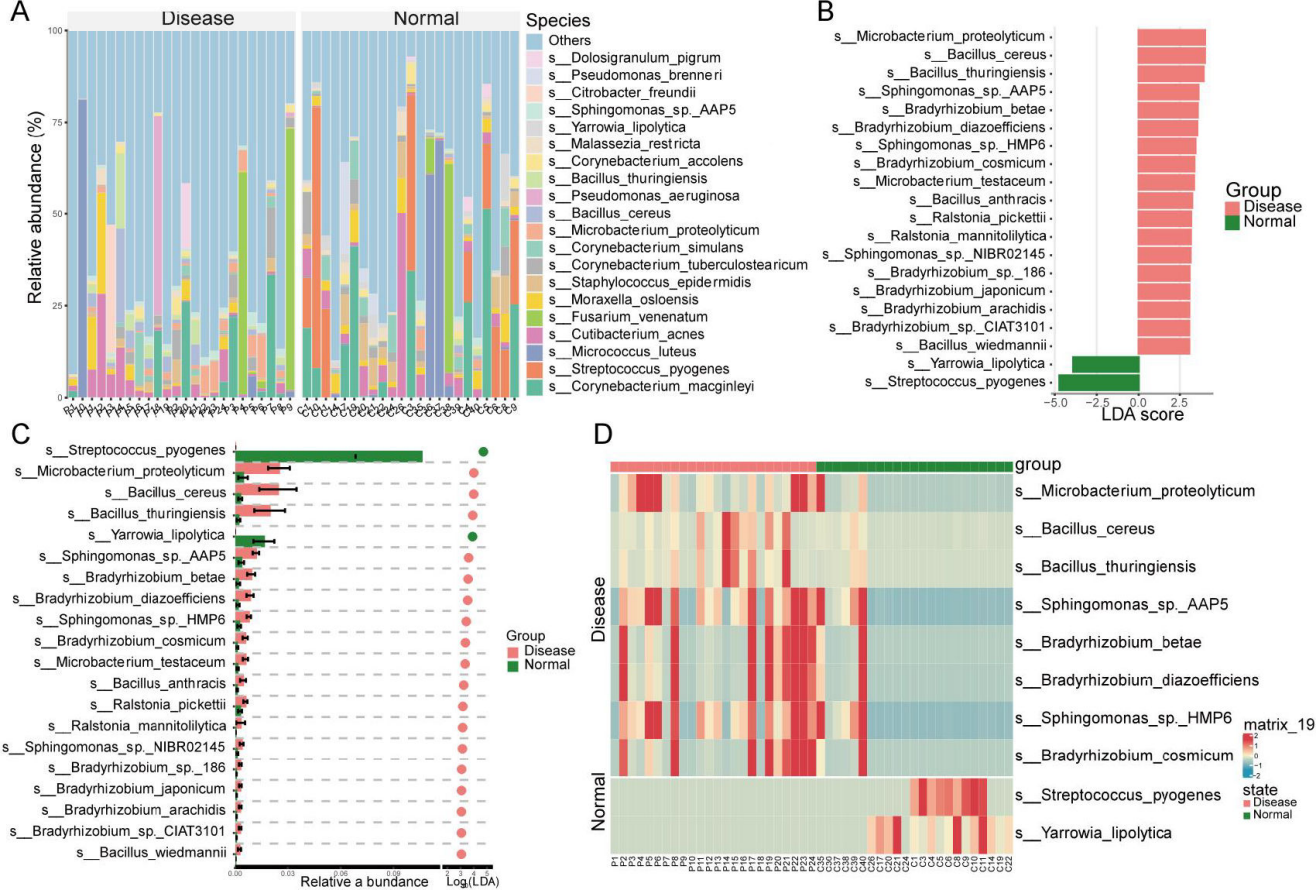
Species-level microbial abundance and differences between the Disease and Normal groups Species with higher abundance in the Disease and Normal groups. (**A**) Species with higher abundance in the Disease group compared with the Normal group. (**B and C**) Potential biomarkers and differentially abundant species between the Disease and Normal groups. (**D**) Heatmap showing the top 10 differentially abundant species with the highest relative abundance across all samples in both groups.

At the species level, 9,724 bacterial species were identified across the Disease and Normal groups. Figure 7A presents the 20 most abundant species. The Disease group was dominated by *Corynebacterium macginleyi* and *Cutibacterium acnes*, whereas the Normal group primarily consisted of *C. macginleyi*, *Streptococcus pyogenes*, *Micrococcus luteus*, and *C. acnes*. Other abundant species included *Fusarium venenatum*, *Moraxella osloensis,* and *Staphylococcus epidermidis,* and so on (Fig. 7A).

Differential and LEfSe analyses identified 20 potential biomarkers. Compared with the Normal group, 18 species, including *Microbacterium proteolyticum*, *Bacillus cereus*, and *Bacillus thuringiensis*, were significantly enriched in the Disease group. Conversely, *S. pyogenes* and *Yarrowia lipolytica* were significantly more abundant in the Normal group (Fig. 7B and C). Heatmap analysis further confirmed that *S. pyogenes* and *Y. lipolytica* were elevated in the Normal group, while Bacillus cereus and others were enriched in the disease group (Fig. 7D).

### Species-level composition of the ocular surface microbiome in disease and tissue groups

**Fig 8:**
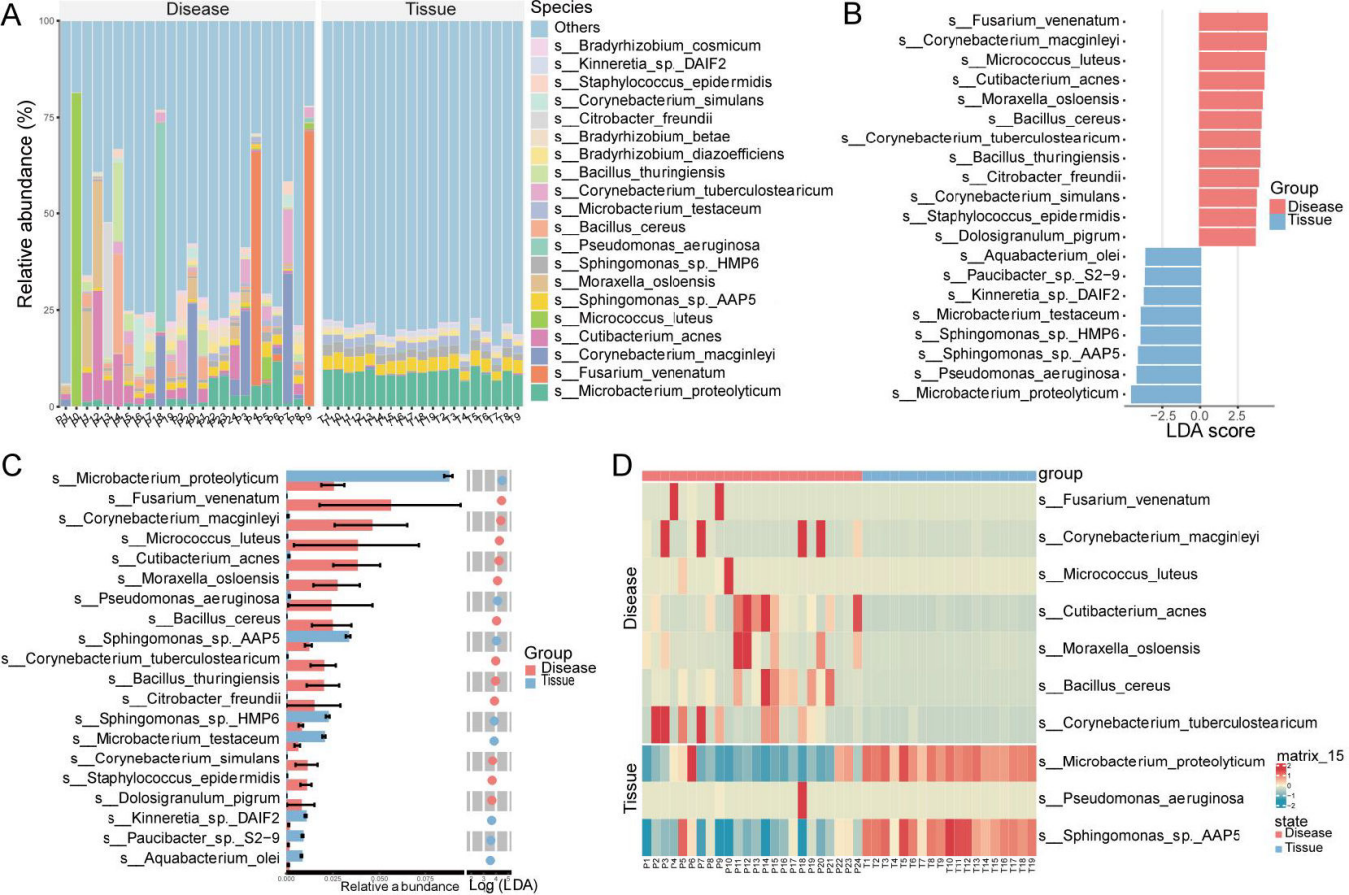
Species-level microbial abundance and differences between the Disease and Tissue groups. Species with higher abundance in the Disease and Tissue groups. (**A**) Species with higher abundance in the Disease group compared with the Tissue group. (**B and C**) Potential biomarkers and differentially abundant species between the Disease and Tissue groups. (**D**) Heatmap showing the top 10 differentially abundant species with the highest relative abundance across all samples in both groups.

At the species level, 9,393 bacterial species were identified across the Disease and Tissue groups. Figure 8 presents the 20 most abundant species, with unclassified taxa accounting for a significant proportion in both groups. The tissue group was dominated by *Microbacterium proteolyticum*, *Sphingomonas sp*. AAP5, *Sphingomonas sp*. HMP6, and *Microbacterium testaceum*. Other species included *Bradyrhizobium cosmicum*, *Kinneretia sp*. DAIF2, and *Staphylococcus epidermidis*, among 15 others (Fig. 8A).

Differential and LEfSe analyses identified 20 potential biomarkers. Compared with the Tissue group, 12 species, including *Fusarium venenatum*, *Corynebacterium macginleyi*, and *Micrococcus luteus*, were significantly enriched in the Disease group. Conversely, eight species, such as *Microbacterium proteolyticum* and *Pseudomonas aeruginosa*, were more abundant in the Tissue group (Fig. 8B and C). Heatmap analysis further confirmed the increased abundance of *Microbacterium proteolyticum*, *P. aeruginosa*, and *Sphingomonas* in the Tissue group, while *F. venenatum*, *C. macginleyi*, and five other species were elevated in the Disease group (Fig. 8D). Notably, a substantial proportion of sequences remained unclassified at the species level, highlighting the high microbial diversity within these samples.

### Viral composition analysis

**Fig 9:**
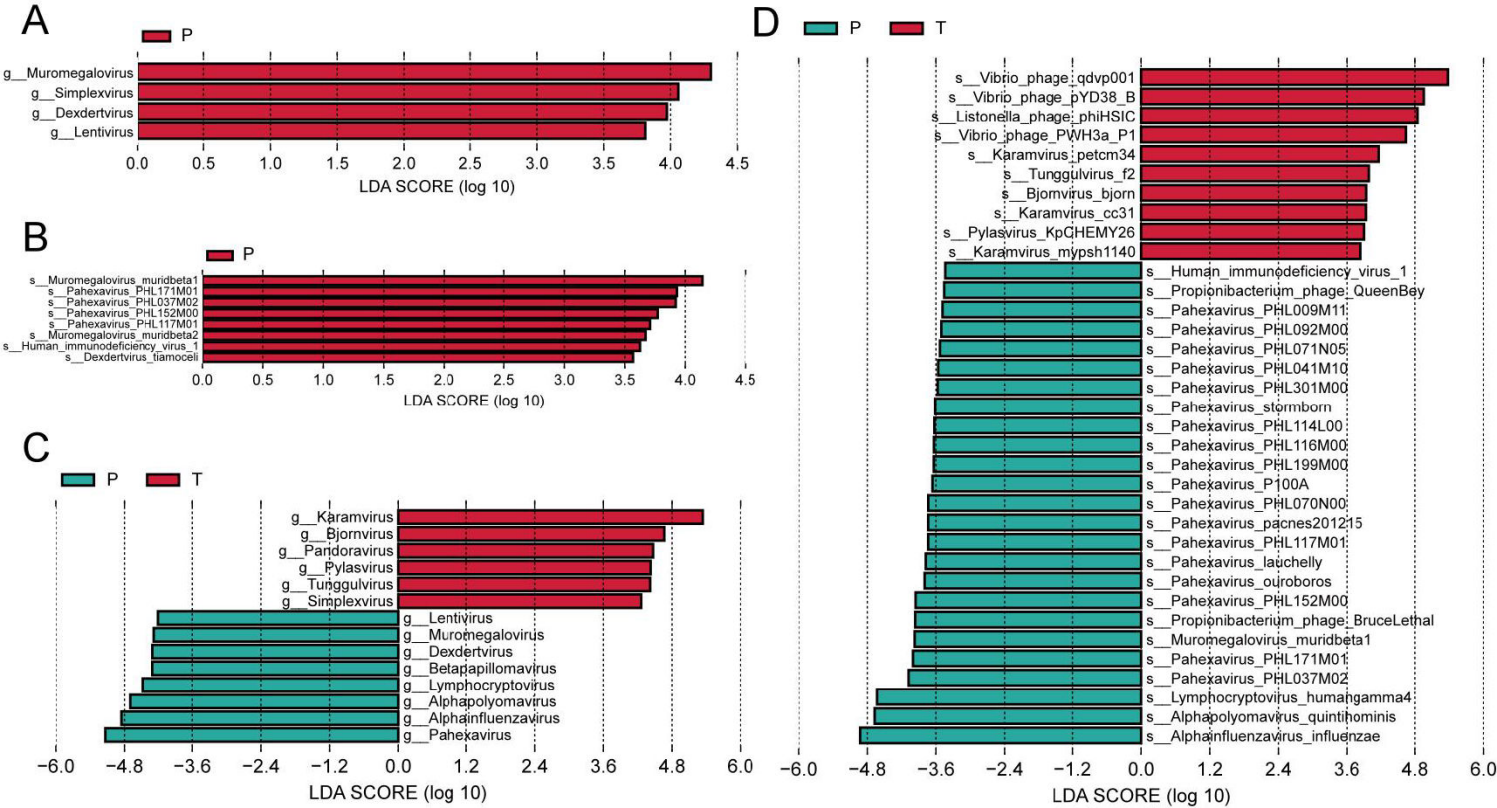
Differential viral analysis at the genus and species levels among the three groups. (**A**) Genus-level and (**B**) species-level differential viruses between the ocular surface of pterygium patients (Disease, *P* group) and healthy controls (Normal, C group). (**C**) Genus-level and (**D**) species-level differential viruses between the ocular surface (Disease, *P* group) and pterygium tissue (Tissue, T group).

To investigate the potential association between pterygium and viruses, we analyzed viral components within the microbiome. Since viruses constituted a minimal fraction of the total microbiota (Fig. 3), their relative abundance was calculated based on the total viral counts in each group. The result revealed that only four viral genera (Lentivirus, Dexdertvirus, Simplexvirus, and Muromegalovirus) were significantly enriched in the Disease group compared with the Normal group (Fig. 9A), with eight species, including Muromegalovirus muridbeta1 and Pahexavirus PHL171M01, exhibiting higher abundance (Fig. 9B). Comparison between the Disease and Tissue groups showed that the Disease group was enriched in Pahexavirus, Alphainfluenzavirus, and Alphapolyomavirus, whereas the Tissue group had a higher prevalence of Karamvirus, Bjornvirus, and Pandoravirus (Fig. 9C). At the species level, Alphainfluenzavirus influenzae, Alphapolyomavirus quintihominis, and Lymphocryptovirus humangamma4 were predominant in the Disease group, while Vibrio phage qdvp001, Vibrio phage pYD38-B, and Listonella phage phiHSIC were enriched in the Tissue group (Fig. 9D).

Interestingly, because we found that many *Vibrio* phages were enriched in tissues, and phages have strong specificity, we went back to analyze the distribution of *Vibrio* bacteria in different samples and found that *Vibrio diabolicus* played a major role. *Vibrio diabolicus* was found in nine tissue samples, which was significantly different from the healthy group and the ocular surface group (Table 2; Fig. S3). Among these nine samples, six samples were annotated to *Vibrio* phage qdvp001, four samples were annotated to *Vibrio* phage pYD38-B, and no corresponding phages were found in tissues that were not annotated to *Vibrio diabolicus*. This further verified the result that multiple high-abundance *Vibrio* phages were annotated in tissues.

**TABLE 2:** Ppm of *Vibrio diabolicus*, *Vibrio* phage qdvp001, and *Vibrio* phage pYD38-B

ID	Location	*Vibrio diabolicus* (ppm)	*Vibrio* phage qdvp001 (ppm)	*Vibrio* phage pYD38-B (ppm)
T1	Wenzhou, Zhejiang, China	4.426733252	0	0
T3	Wenzhou, Zhejiang, China	4.515089423	2.215436494	2.664397455
T4	Taizhou, Zhejiang, China	5.315238875	3.735761635	0
T6	Wenzhou, Zhejiang, China	4.703842705	2.279649598	2.279649598
T13	Wenzhou, Zhejiang, China	4.633673274	2.374434762	0
T14	Wenzhou, Zhejiang, China	5.163215705	0	2.489650224
T15	Wenzhou, Zhejiang, China	5.650527772	3.3948076	3.09573358
T16	Fuding, Fujian, China	4.56308397	2.900765247	0
T18	Taizhou, Zhejiang, China	2.784656507	0	0
T2	Lanxi, Zhejiang, China	0	0	0
T5	Wenzhou, Zhejiang, China	0	0	0
T7	Honghu, Wuhan, China	0	0	0
T8	Jinhua, Zhejiang, China	0	0	0
T9	Wenzhou, Zhejiang, China	0	0	0
T10	Wenzhou, Zhejiang, China	0	0	0
T11	Fuding, Fujian, China	0	0	0
T12	Wenzhou, Zhejiang, China	0	0	0
T17	Wenzhou, Zhejiang, China	0	0	0
T19	Wenzhou, Zhejiang, China	0	0	0

### COG functional annotation and differential functional analysis

**Fig 10:**
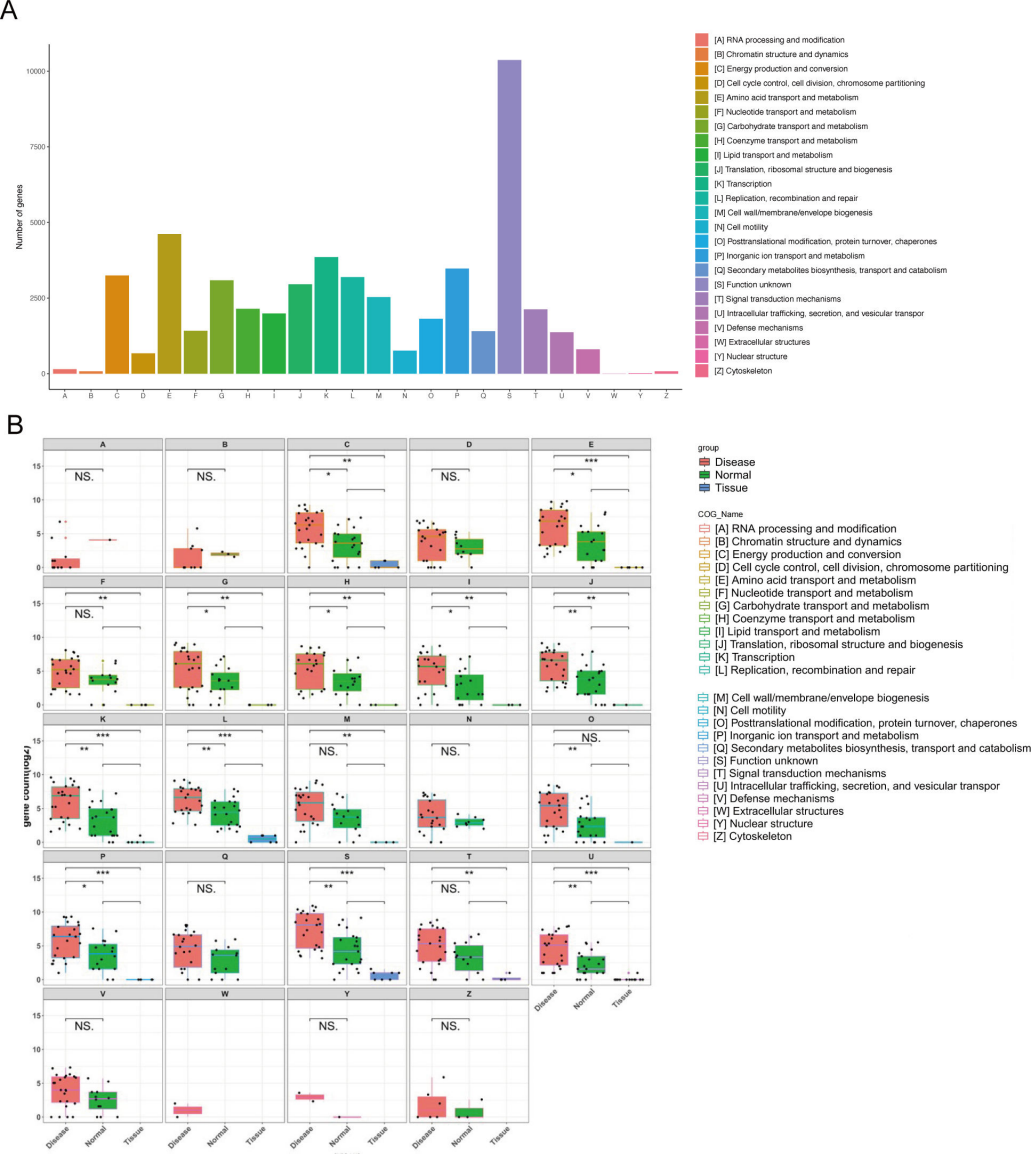
COG classification of the microbiome across the three groups. (**A**) Overall COG functional classification in the Disease, Tissue, and Normal groups. (**B**) Differential COG functional classifications between the Disease and Tissue groups and between the Disease and Normal groups.

Comparison of the non-redundant gene sets from the Disease, Tissue, and Normal groups with the COG database revealed that gene functions primarily clustered in energy production and conversion, amino acid transport and metabolism, nucleotide transport and metabolism, transcription, and inorganic ion transport and metabolism (Fig. 10A). Further analysis identified 12 COG functional categories differing between the Disease and Normal groups, including energy production and conversion, amino acid transport and metabolism, and carbohydrate transport and metabolism. Additionally, 14 functional categories varied between the Disease and Tissue groups, encompassing energy production and conversion, amino acid transport and metabolism, and nucleotide transport and metabolism (Fig. 10B).

### KEGG and CAZy functional annotation

**Fig 11:**
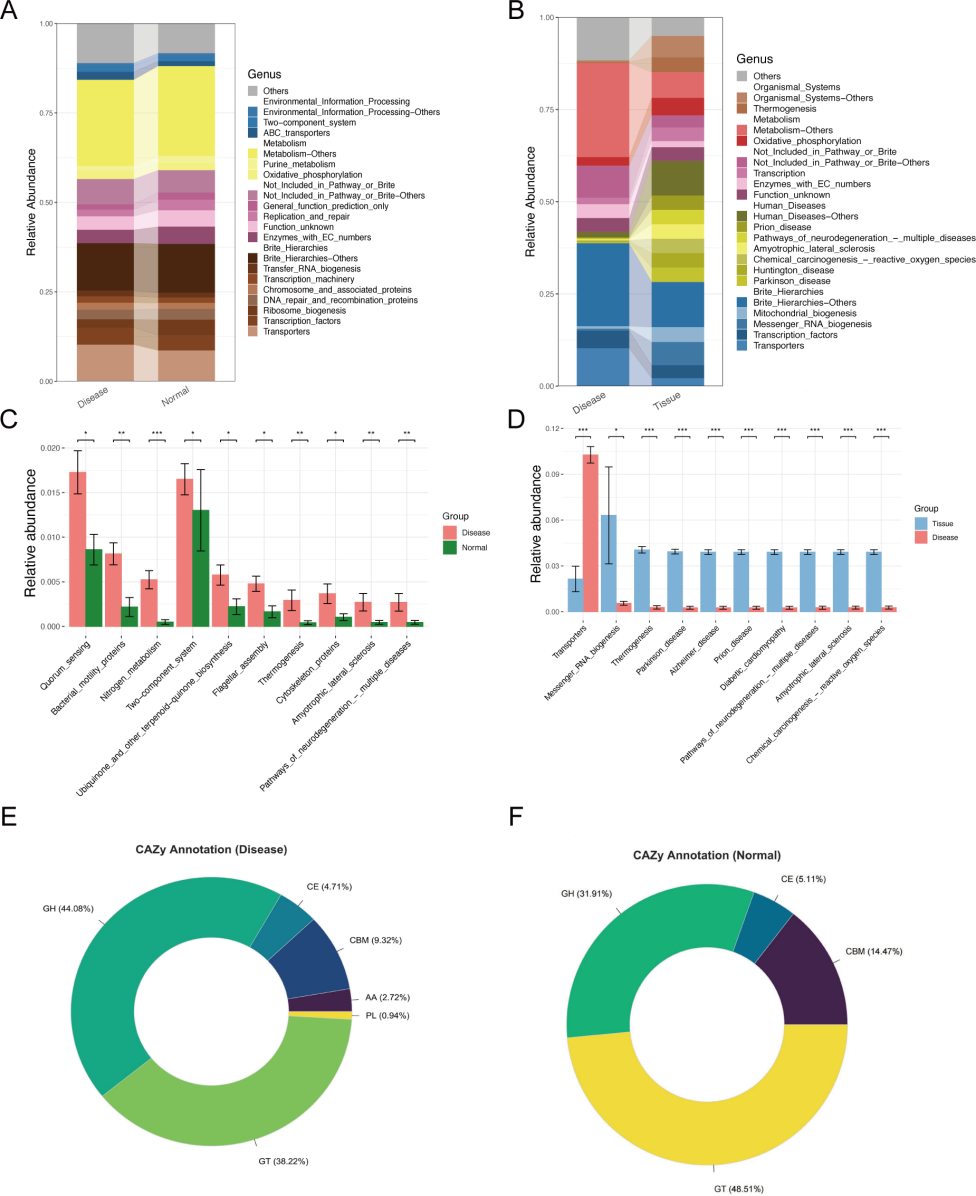
KEGG metabolic pathways and CAZy carbohydrate enzyme composition across the three groups. (**A and C**) Differential KEGG functional classifications between the Disease and Normal groups. (**B and D**) Differential KEGG functional classifications between the Disease and Tissue groups. (**E**) CAZy enzyme composition annotated in the Disease group. (**F**) CAZy enzyme composition annotated in the Normal group.

The KEGG metabolic pathways in the Normal and Disease groups were largely similar, primarily involving metabolism, transport proteins, general function prediction, and Brite Hierarchies (Fig. 11A). Differential analysis identified 10 microbial-associated pathways with significant differences between the two groups (Fig. 11C). In contrast, the Tissue group exhibited substantial metabolic pathway alterations, mainly related to human diseases, Brite Hierarchies−Others, and metabolism (Fig. 11B). Further analysis revealed 10 disease-associated pathways significantly enriched in the Tissue group, including RNA biosynthesis, thermogenesis, Parkinson’s disease, Alzheimer’s disease, prion disease, diabetic cardiomyopathy, neurodegenerative disorders, amyotrophic lateral sclerosis, and chemical carcinogenesis—reactive oxygen species (Fig. 11D).

CAZy analysis identified 438 metabolism-related enzymes with functional annotations. No enzyme-related pathways were detected in the Tissue group, suggesting that pterygium tissue may not participate in ocular surface microbial competition. In both the Disease and Normal groups, functional annotations were dominated by “GHs” and “GTs.” However, the Disease group exhibited significantly higher levels of “PLs” and “AAs,” which may be closely associated with pterygium progression (Fig. 11E and F).

### Virulence and antibiotic resistance genes (ARGs) in the ocular surface microbiome

**Fig 12:**
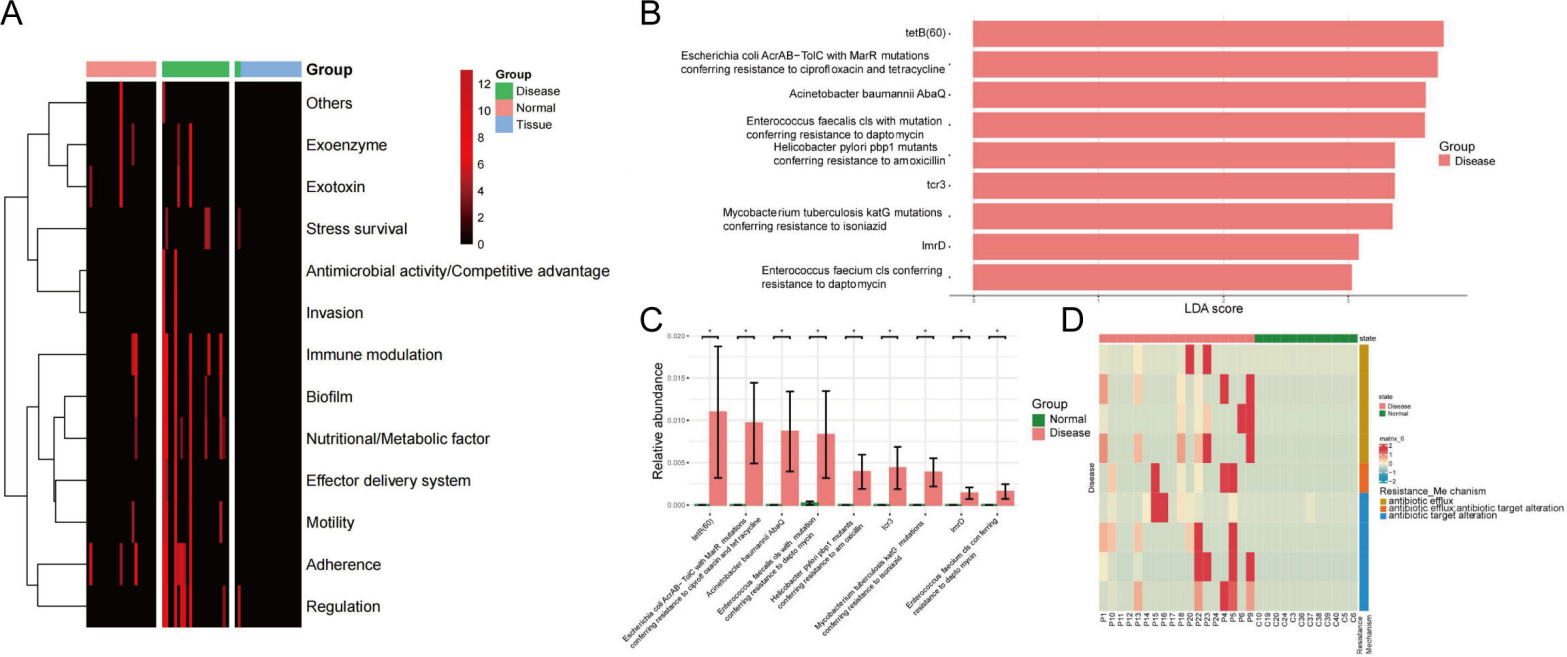
Virulence factors and antibiotic resistance genes across the three groups. (**A**) Heatmap showing the relative abundance of VF functional categories in the three groups. (**B**) Potential biomarkers identified in the Disease group. (**C**) Differential antibiotic resistance mechanisms between the Disease and Normal groups. (**D**) Heatmap illustrating the relative abundance of resistance mechanisms in the Disease and Normal groups.

A total of 13 potential virulence functions were identified in both the Disease and Normal groups, with a significant shift in virulence factor (VF) composition in the Disease group compared with the Normal group (Fig. 12A). In the Disease group, VFs were primarily associated with immune modulation, biofilm formation, nutrient acquisition/metabolism, motility, adhesion, and regulation, whereas the Normal group mainly exhibited immune modulation and adhesion-dependent factors. Notably, VF enrichment in the Normal group suggests a potential role in inhibiting pathogenic bacteria. No virulence factors were detected in the pterygium tissue (Tissue group), indicating a lack of infection-inducing capacity.

A total of 52 ARGs were detected across all samples, with significant compositional differences between the Disease and Normal groups. LEfSe analysis (LDA >3, Fig. 12B) identified nine potential biomarkers in the Disease group. Figure 12C highlights differentially abundant ARGs, including the tetracycline resistance gene tetB (50) and *Escherichia coli* AcrAB-TolC carrying MarR mutations, which confer resistance to ciprofloxacin and tetracycline. Heatmap analysis further revealed that antibiotic resistance mechanisms in the Disease group were mainly linked to efflux pumps, target modifications, and their combined effects. Notably, no ARGs were detected in the pterygium tissue samples (Fig. 12D).

## DISCUSSION

The human ocular surface harbors a diverse range of microorganisms, including bacteria, viruses, and fungi, which are closely associated with ocular physiology, immune responses, and disease development (6, 18, 51–55). This study applied metagenomic shotgun sequencing and bioinformatics analysis to compare the microbiomes of conjunctival sac secretions from pterygium patients (Disease group) and healthy controls (Normal group), as well as pterygium tissue (Tissue group). We propose that simultaneous analysis of the ocular surface and tissue microbiomes can provide complementary insights. The ocular surface microbiome reflects host-environment interactions relevant to pterygium pathogenesis, whereas the tissue microbiome, despite perturbations by disinfectants, suggests the presence of unique microbial signatures at the local lesion site. To our knowledge, this is the first study to compare the microbiomes of these three groups. The results showed a significant increase in microbial diversity and dysbiosis in the Disease group, which may be linked to pterygium progression. Functional prediction analysis indicated an upregulation of genes related to immune regulation and cellular damage repair in this group (51). Furthermore, significant differences between the Disease and Tissue groups suggest that pterygium tissue harbors a distinct microbiome, providing important insights into its pathogenesis and potential therapeutic strategies (56).

Further analysis revealed that the Shannon and inverse Simpson indices were significantly higher in the Disease group than in the Normal group, while the Shannon index was notably lower in the Tissue group compared with the Disease group. Although β-diversity did not differ significantly between the Normal and Disease groups, a significant variation was observed between the Disease and Tissue groups. These findings suggest that the ocular microbiome undergoes substantial alterations in pterygium tissue, potentially contributing to disease pathogenesis. Of note, the reduced microbial diversity observed in tissue samples may partly reflect the influence of preoperative antisepsis.

The richness and diversity of ocular surface microbiota may reflect environmental exposure (57). This study identified *Bacteroidetes* and *Actinobacteria* as the dominant phyla of ocular surface bacterial colonies, consistent with previous research (58–60). *Corynebacterium*, *Staphylococcus*, and *Streptococcus* were more prevalent in the Normal group, reinforcing their role as core components of the ocular microbiome (50). At the species level, the most abundant taxa in the Normal group included *Streptococcus pyogenes* and *Corynebacterium macginleyi*, both common pathogens (61), suggesting the presence of physiological mechanisms that suppress pathogenic bacteria. However, microbial dysbiosis may enhance pathogen virulence, increasing the risk of infection (9). In the Disease group, *Bacillus* and *Bacillus cereus*, identified as potential biomarkers, have previously been recognized as important ocular pathogens (62, 63). The enrichment of *Microbacterium*, *Sphingomonas*, and *Pseudomonas* in tissues indicates that they may invade pterygium tissue, causing the progression and occurrence of pterygium. Also noteworthy is the highly enriched presence of multiple *Vibrio* phages and *Vibrio diabolicus* in tissues. *Vibrio diabolicus* can invade various tissue cells, and many studies have shown that it is primarily associated with marine organisms, with infection primarily found in coastal areas (64, 65). Our tracing of the source revealed that all patients with high abundance of *Vibrio* phages and *Vibrio diabolicus* in their tissues were from coastal areas. Therefore, we speculate that one of the potential pathogens of pterygium in coastal areas is lesions caused by infection with *Vibrio diabolicus*.

KEGG functional annotation revealed a significant increase in genes related to bacterial motility proteins, nitrogen metabolism, two-component systems, and chemotaxis in the Disease group (66, 67), suggesting their involvement in pterygium progression. Additionally, genes associated with lipid transport and metabolism, translation, and replication were significantly enriched in the Disease group, whereas those linked to energy production, amino acid transport, and metabolism were more abundant in the Tissue group. As a key tear film component, lipids play a crucial role in ocular surface disease treatment (68–71), but whether the ocular microbiome contributes to lipid metabolism remains unclear.

COG annotation showed that unknown functions were predominant in the Disease group, followed by amino acid transport and metabolism pathways. The high abundance of these pathways may be attributed to *Corynebacterium*, suggesting its potential role in pterygium (72). Moreover, genes related to “energy production and conversion,” “nucleotide transport and metabolism,” and “inorganic ion transport and metabolism” were significantly enriched. As a major anionic lipid in the eye (73, 74), sphingomyelin interacts with divalent cations, potentially influencing tear film stability (75, 76). Investigating how the ocular microbiome modulates tear film stability through ion transport and metabolism could aid in developing novel therapeutic strategies. Notably, genes linked to transport proteins, thermogenesis, and human diseases (e.g., Parkinson’s) were significantly upregulated in the Tissue group, implying a potential role in counteracting metabolic disorders through thermogenesis, though the underlying mechanisms require further investigation (77, 78). This cross-system signal suggests that ocular surface microecological imbalance may not only affect local areas, but may also have potential links with systemic diseases through inflammatory and metabolic pathways.

This study identified marked differences in VF and ARG profiles between the Disease and Normal cohorts, suggesting a potential role of ocular surface microbiota in pterygium development. In the Disease group, VFs related to immune regulation, biofilm formation, adhesion, and motility were significantly enriched, indicating enhanced microbial colonization capabilities and host-microbe interactions (79, 80). In contrast, VF enrichment patterns observed in the Normal group may reflect inherent protective mechanisms against pathogenic colonization. Importantly, no detectable VFs were found in pterygium tissues, implying minimal infection risk and supporting the hypothesis that microbiota imbalance influences disease progression through indirect pathways rather than direct infection. ARG analysis revealed a higher abundance of resistance genes in the Disease group, including tetB (50) and MarR-mutated *E. coli* AcrAB-TolC associated with tetracycline and fluoroquinolone resistance. Mechanistically, antibiotic efflux, target modification, and their synergistic effects emerged as key resistance strategies, potentially conferring survival advantages under selective pressure (81). In addition, the distinct ARG profiles observed between diseased and healthy eyes suggest that microbiome surveillance could play an important role in guiding perioperative infection control and antimicrobial stewardship. Notably, the absence of ARGs in pterygium tissues suggests that resistance determinants likely originate from ocular surface microbiota rather than intra-tissue colonization, which further emphasizes the importance of preoperative ocular surface microecological management (preoperative disinfection measures).

This study has several limitations. First, the sample size was relatively small, which may limit the generalizability of our findings. This constraint primarily reflects the strict inclusion criteria and the inherent challenges of obtaining paired ocular surface and tissue samples. Second, our methodology and cross-sectional design can only reveal associations between microbial dysbiosis and pterygium but cannot establish causality. Longitudinal investigations and experimental models will be required to determine whether microbial alterations act as a cause or consequence of the disease. Third, the absence of detectable microbes in excised pterygium tissue may partly result from the use of preoperative antiseptic agents, which could have reduced microbial load independently of biological differences. Finally, this was a single-center study; therefore, external validation in geographically diverse, independent cohorts is essential to confirm the robustness and reproducibility of our results.

Future research should aim to overcome these limitations and further expand our understanding of the ocular microbiome in pterygium. Large-scale, multicenter studies with diverse populations are needed to validate the microbial signatures observed in this cohort and to enhance the generalizability of the findings. Longitudinal studies and experimental models will be critical to elucidate whether specific microbial alterations drive or result from disease progression. From a translational perspective, microbiome profiling may enable risk stratification for the occurrence and recurrence of pterygium, guide microbiome-targeted therapeutic interventions, and inform personalized preventive strategies to complement surgical treatment, for example, regional prevention of *Vibrio*, antimicrobial treatments for different ARG spectrums.

### Conclusion

This metagenomic study systematically profiled the ocular surface and tissue microbiomes in pterygium reveal marked differences in microbial diversity, taxonomic composition, and functional potential between diseased and healthy eyes. The enrichment of *Microbacterium proteolyticum*, together with its even higher abundance in tissue samples, suggests its value as a potential biomarker for pterygium. Distinct functional pathways—including bacterial motility, chemotaxis, biofilm formation, and immune regulation—were upregulated in diseased eyes, along with multiple antibiotic resistance determinants. In contrast, tissue microbiomes were characterized by reduced microbial richness, enrichment of viral species, such as *Vibrio* phages, and pathways linked to human systemic diseases, but no resistance genes. Importantly, the predominance of *Vibrio* phages and their bacterial host *Vibrio diabolicus* in tissue samples indicates a potential region-specific microbial hazard. Together, these results provide novel evidence that microbial dysbiosis may contribute to pterygium development and recurrence risk. Future studies with larger cohorts and longitudinal designs are warranted to clarify causal relationships and explore microbiome-targeted strategies as adjuncts to surgical treatment.

## Data Availability

The sequencing data of this study have been uploaded to NCBI with accession numbers SRR33484664 to SRR33484682, SRR33514152 to SRR33514175, and SRR33512234 to SRR33512239 in PRJNA1261000.

## SUPPLEMENTAL MATERIAL

Figure S1 spectrum.01730-25-s0001.pdf β-diversity between the Disease group and the Normal group.

Figure S2 spectrum.01730-25-s0002.pdf β diversity between the Disease group and the Tissue group.

Figure S3 spectrum.01730-25-s0003.pdf Statistical analysis of the abundance of *Vibrio diabolicus* (A), *Vibrio* phage qdvp001 (B), and *Vibrio* phage pYD38.B (C)

Table S1 spectrum.01730-25-s0004.xlsx Basic information of subjects.

Table S2 spectrum.01730-25-s0005.xlsx Annotation information of species relative abundance in healthy ocular surface, diseased ocular surface, and tissue samples.

Supplementary figure legend spectrum.01730-25-s0006.docx Legends for Figures S1 to S3.

ASM does not own the copyrights to Supplemental Material that may be linked to, or accessed through, an article. The authors have granted ASM a non-exclusive, world-wide license to publish the Supplemental Material files. Please contact the corresponding author directly for reuse.
