# Supplementary figures and images for: Exploring the ocular microecology and its role in pterygium based on metagenomics
Source: Microbiol Spectr. 2025 Oct 13;13(11):e01730-25. doi: 10.1128/spectrum.01730-25 (PMC12584759; doi:10.1128/spectrum.01730-25)

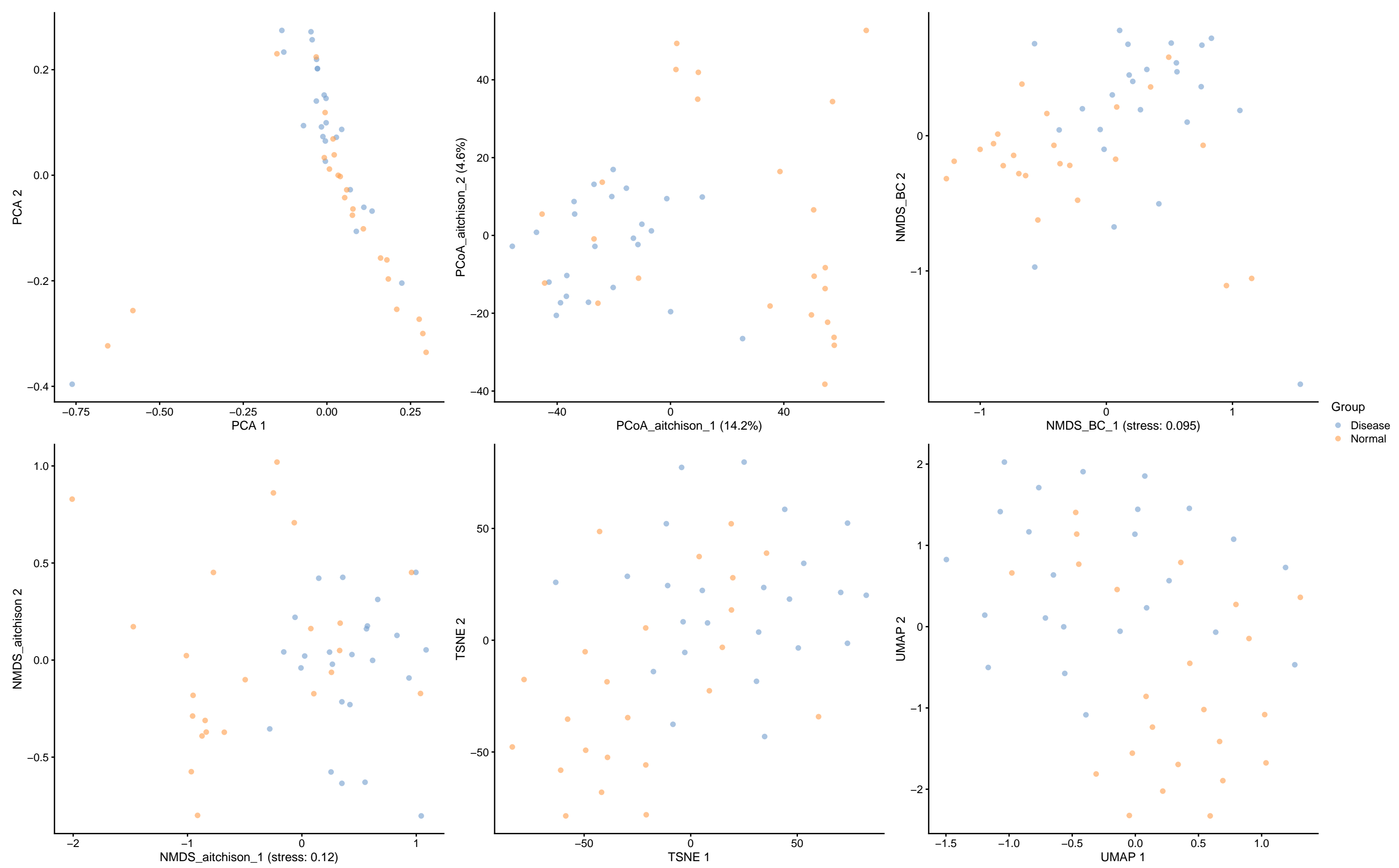

Supplement: Figure S1 — β-diversity between the Disease group and the Normal group. [file spectrum.01730-25-s0001.pdf]

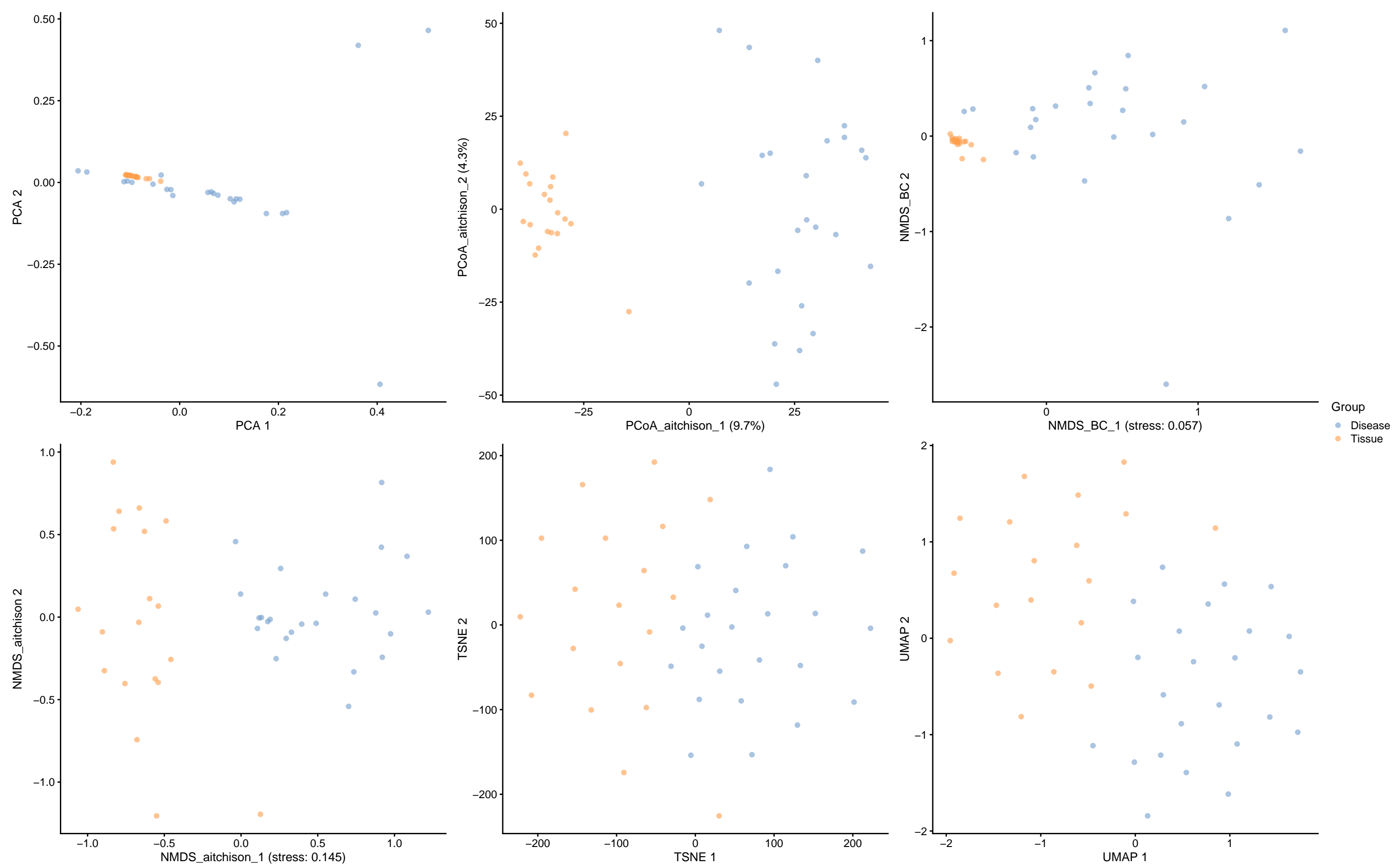

Supplement: Figure S2 — β diversity between the Disease group and the Tissue group. [file spectrum.01730-25-s0002.pdf]

A

Anova Test Pvalue:  $1.43 \times 10^{-5}$ 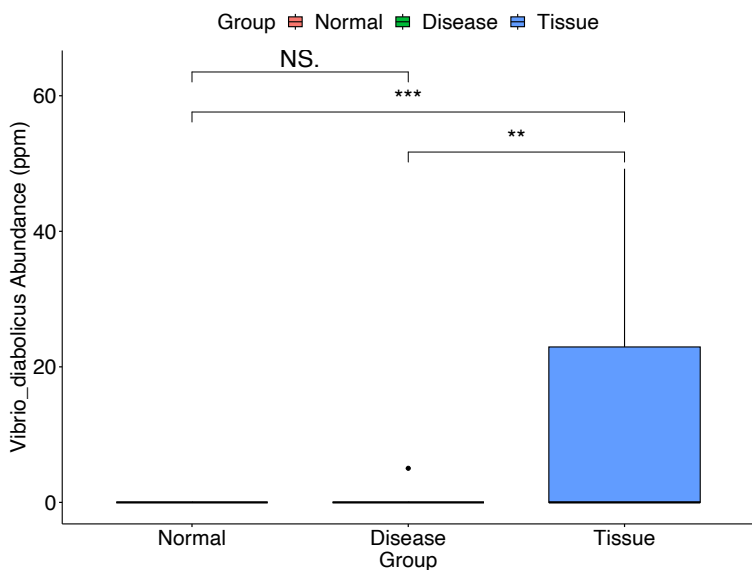

B

Anova Test Pvalue: 0.00153

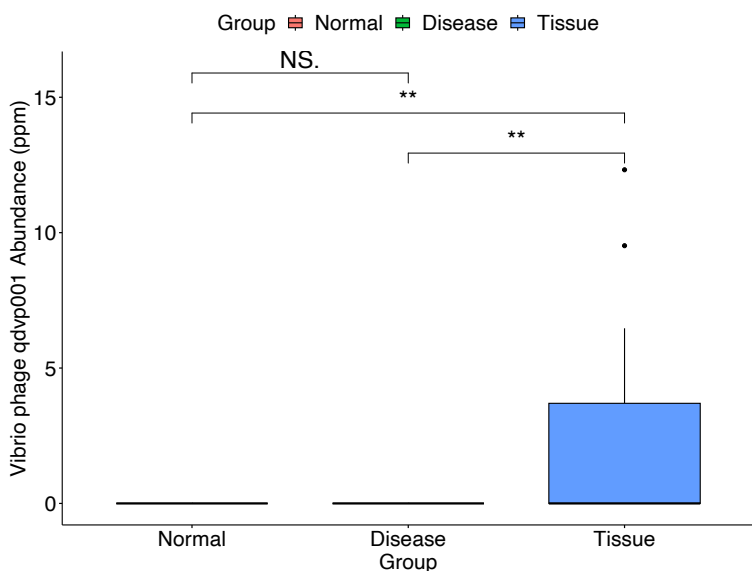

C

Anova Test Pvalue: 0.00822

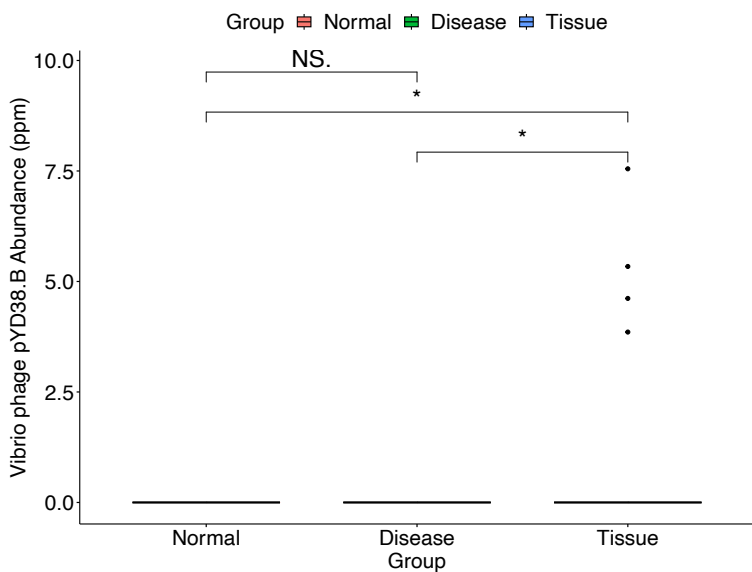

Supplement: Figure S3 — Statistical analysis of the abundance of Vibrio diabolicus (A), Vibrio phage qdvp001 (B), and Vibrio phage pYD38.B (C) [file spectrum.01730-25-s0003.pdf]
