# Supplementary material for: Exploring the ocular microecology and its role in pterygium based on metagenomics
Source: Microbiol Spectr. 2025 Oct 13;13(11):e01730-25. doi: 10.1128/spectrum.01730-25 (PMC12584759; doi:10.1128/spectrum.01730-25)
Supplement: Supplementary figure legend — Legends for Figures S1 to S3. [file spectrum.01730-25-s0006.docx]

**Supplemental Figure 1.** β diversity between the Disease group and the Normal group

**Supplemental Figure 2.** β diversity between the Disease group and the Tissue group

**Supplemental Figure 3.**: Statistical analysis of the abundance of *Vibrio diabolicus* (A), Vibrio phage qdvp001 (B), and Vibrio phage pYD38.B (C). *: p < 0.05, **: p < 0.01, ***: p < 0.001
